# Supplementary material for: Cardiotoxicity Associated with Gemcitabine: Literature Review and a Pharmacovigilance Study
Source: Pharmaceuticals (Basel). 2020 Oct 21;13(10):325. doi: 10.3390/ph13100325 (PMC7594046; doi:10.3390/ph13100325)

**SUPPLEMENTARY MATERIAL**

**Supplementary Table S1.** Concurrent adverse events in individual case safety reports of gemcitabine-associated cardiovascular adverse drug reactions (CV-ADRs) in VigiBase (accessed on 1 April 2019)

| **Concurrent adverse events** | | **Myocardial ischaemia**  **n=119** | **Supraventricular arrhythmias**  **n=308** | **Pericardial diseases**  **n=164** | **Heart failure**  **n=484** | ***p*** |
| --- | --- | --- | --- | --- | --- | --- |
| None | | 52/119 (44%) | 64/308 (21%) | 22/164 (13%) | 114/484 (23%) | **<0.0001** |
| Cardiovascular disorders | | | | | | |
|  | Myocardial infarction |  | 4/308 (1%) | 0/164 (0%) | 5/484 (1%) | 0.36 |
|  | SVA | 4/119 (3%) |  | 8/164 (5%) | 36/484 (7%) | 0.18 |
|  | Pericardial diseases | 0/119 (0%) | 8/308 (3%) |  | **33/484 (7%)** | **<0.001** |
|  | Heart failure | 5/119 (4%) | 36/308 (12%) | **33/164 (20%)** |  | **<0.001** |
|  | Capillary leak syndrome | 1/119 (1%) | 0/308 (0%) | **4/164 (2%)** | 4/484 (1%) | **0.05** |
|  | Thrombotic microangiopathy | 1/119 (1%) | 1/308 (0%) | **6/164 (4%)** | 14/484 (3%) | **0.05** |
|  | Hypertension | 4/119 (3%) | 12/308 (4%) | 10/164 (6%) | 33/484 (7%) | 0.23 |
| Gastrointestinal disorders | | | | | | |
|  | Ascites | 1/119 (1%) | 1/308 (0%) | **8/164 (5%)** | 4/484 (1%) | **<0.001** |
|  | Elevations in liver function tests | 5/119 (4%) | 18/308 (6%) | 7/164 (4%) | 22/484 (4%) | 0.80 |
|  | Nausea or vomiting | 9/119 (8%) | **45/308 (15%)** | 20/164 (12%) | 31/484 (7%) | **0.001** |
|  | Diarrhea | 4/119 (3%) | 14/308 (5%) | 3/164 (2%) | 19/484 (4%) | 0.51 |
| Endocrino-metabolic disorders | | | | | | |
|  | Potassium imbalance | 4/119 (3%) | **17/308 (6%)** | **11/164 (6%)** | 9/484 (2%) | **0.01** |
|  | Albumin or protein decreased | 2/119 (2%) | 2/308 (1%) | **7/164 (4%)** | 14/484 (3%) | 0.06 |
|  | Thyroid disorders | 0/119 (0%) | 0/308 (0%) | **2/164 (2%)** | 0/484 (0%) | **0.01** |
| Pulmonary disorders | | | | | | |
|  | Interstitial lung disease | 2/119 (2%) | 8/308 (3%) | 5/164 (3%) | 15/484 (3%) | 0.85 |
|  | Pleural effusion | 4/119 (3%) | 20/308 (7%) | **56/164 (34%)** | 65/484 (13%) | **<0.0001** |
|  | Pneumonitis or pneumonia | 4/119 (3%) | **48/308 (16%)** | 15/164 (9%) | 61/484 (13%) | **<0.0001** |
|  | Pulmonary embolism | 7/119 (6%) | 12/308 (4%) | 4/164 (3%) | 16/484 (3%) | 0.46 |
|  | Respiratory failure or distress | 6/119 (5%) | 20/308 (7%) | 9/164 (5%) | 33/484 (7%) | 0.86 |
| Urological disorders | | | | | | |
|  | Renal failure or injury | 7/119 (6%) | 22/308 (7%) | 16/164 (10%) | **70/484 (14%)** | **<0.01** |
|  | Proteinuria & nephrotic syndrome | 2/119 (2%) | 4/308 (1%) | 2/164 (2%) | 10/484 (2%) | 0.82 |
|  | Hematuria | 1/119 (1%) | 0/308 (0%) | 0/164 (0%) | 5/484 (1%) | 0.19 |
| Haematological disorders | | | | | | |
|  | Anemia | 12/119 (10%) | 46/308 (15%) | 25/164 (15%) | 77/484 (16%) | 0.46 |
|  | Thrombocytopenia | 11/119 (10%) | 38 (12%) | 16/164 (10%) | 55/484 (11%) | 0.75 |
|  | Neutropenia | 6/119 (5%) | 14 (5%) | 6/164 (4%) | 14/484 (3%) | 0.55 |
|  | Pancytopenia | 1/119 (1%) | 7/308 (2%) | 1/164 (1%) | 10/484 (2%) | 0.47 |
|  | Haemolytic uraemic syndrome | 1/119 (1%) | 0/308 (0%) | **6/164 (4%)** | 13/484 (3%) | **0.01** |
| Musculoskeletal disorders | | | | | | |
|  | Back or bone pain | 2/119 (2%) | 7/308 (2%) | 5/164 (3%) | 8/484 (2%) | 0.71 |
|  | Myalgia | 0/119 (0%) | 1/308 (0%) | 0/164 (0%) | 3/484 (1%) | 0.60 |
|  | Arthralgia | 1/119 (1%) | 3/308 (1%) | 1/164 (1%) | 5/484 (1%) | 0.97 |
| Neurological disorders | | | | | | |
|  | Stroke (ischemic or hemorrhagic) | **11/119 (10%)** | 4/308 (1%) | 2/164 (2%) | 10/484 (2%) | **<0.0001** |
|  | Fatigue | 6/119 (5%) | **23/308 (7%)** | 9/164 (5%) | 14/484 (3%) | **0.03** |
| Dermatological disorders | | | | | | |
|  | Oedema | 1/119 (1%) | 17/308 (6%) | **27/164 (16%)** | 56/484 (12%) | **<0.0001** |
|  | Rash | 2/119 (2%) | 5/308 (2%) | 9/164 (5%) | 14/484 (3%) | 0.09 |
| Infections | | | | | | |
|  | Pyrexia | 1/119 (1%) | **27/308 (9%)** | **14/164 (9%)** | 20/484 (4%) | **<0.0001** |
|  | Flu-like symptoms | 0/119 (0%) | 1/308 (0%) | 1/164 (1%) | 3/484 (1%) | 0.80 |
|  | Sepsis (including shock) | 4/119 (3%) | 14/308 (5%) | 3/164 (2%) | 15/484 (3%) | 0.45 |
| Radiation recall reaction | | 0/119 (0%) | 0/308 (0%) | **17/164 (10%)** | 0/484 (0%) | **<0.0001** |
|  | | | | | | |
| Death (any cause) | | 28/119 (24%) | 50/308 (16%) | 32/164 (20%) | 103/484 (21%) | 0.26 |
|  | Death status available | 115/119 (97%) | 289/308 (94%) | 144/164 (88%) | 477/484 (99%) |  |
|  | Cardiovascular adverse drug reactions-related | **20/115 (17%)** | 17/289 (6%) | 5/144 (3%) | **83/477 (17%)** | **<0.0001** |
|  | Cancer progression | 2/115 (2%) | 4/289 (1%) | 4/144 (2%) | 3/477 (1%) | 0.22 |
|  | Other | 2/115 (2%) | 10/289 (3%) | 3/144 (2%) | 10/477 (2%) | 0.61 |

**Supplementary Figure S1.** Summary of one of our case-report presenting a capillary leak syndrome associated with gemcitabine reversible after glucocorticoids.


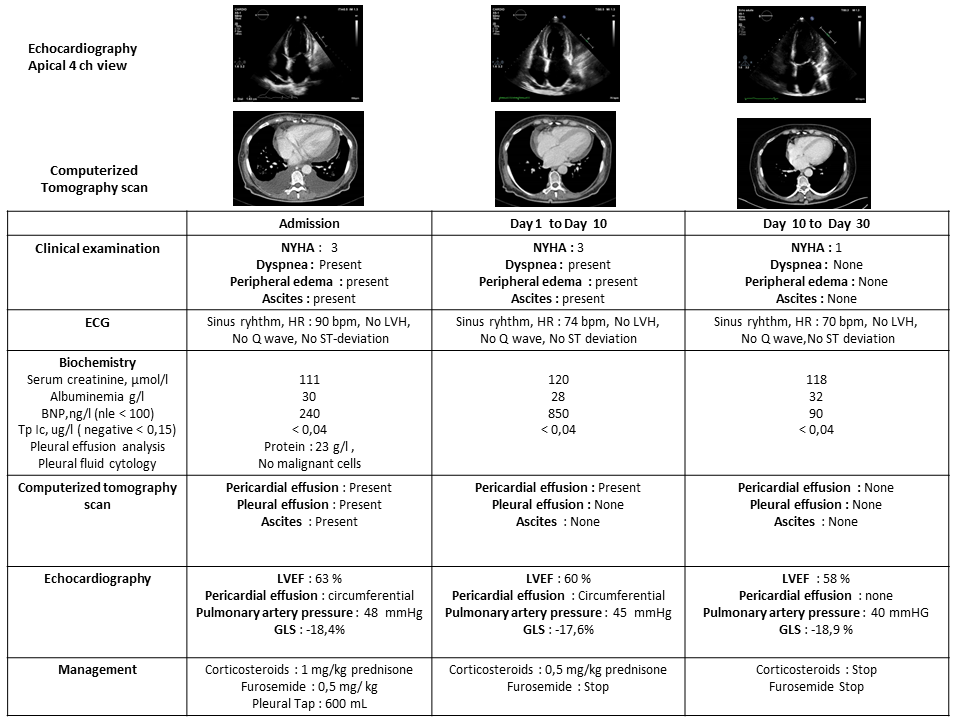


The patient had a metastatic pancreatic carcinoma and the adverse event started 8 months after diagnosis (corresponding to 8 cycles of gemcitabine with a total dose of 24 000mg/m²). As illustrated on the CT-scan images (arterial phase), pleural and pericardial effusion progressively disappeared in less than 10 days after onset.

Abbreviations: BNP : Brain-Type Natriuretic peptide; NYHA : New York Heart Association; GLS : Global Longitudinal Strain; HR: Heart rate; LVEF : Left ventricular ejection fraction; LVH: left ventricular hypertrophy; Tp : Troponin.

**Supplementary Figure S2.** Flowchart of selected studies to describe cardiovascular adverse events associated with gemcitabine.


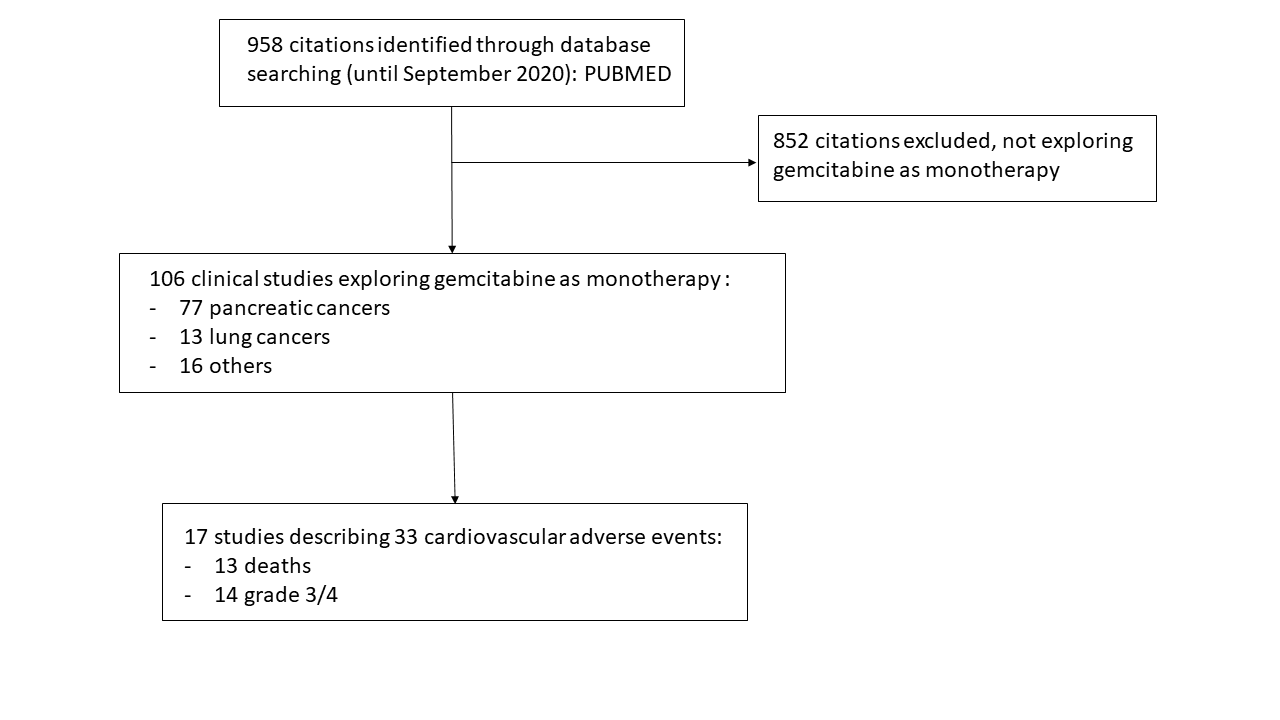


**Supplementary Figure S3.** UpsetR graph representing overlap between cardiovascular sub-classifications.


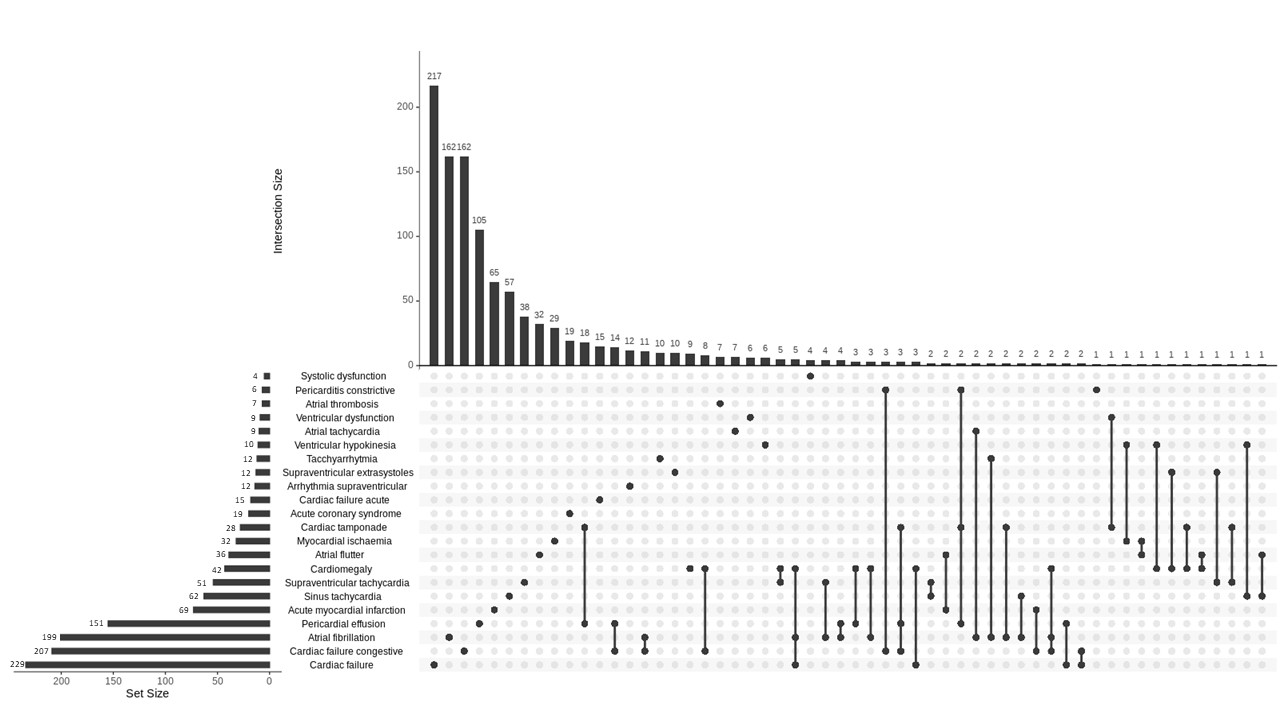

Supplement: Supplementary file 1 [file pharmaceuticals-13-00325-s001.zip › pharmaceuticals-938272-send to proof supplementary MH.docx]
